# Supplementary material for: Engineering the bone metastatic prostate cancer niche through a microphysiological system to report patient-specific treatment response
Source: Commun Biol. 2025 Jul 1;8:961. doi: 10.1038/s42003-025-08384-2 (PMC12218145; doi:10.1038/s42003-025-08384-2)
Supplement: Supplementary file 2 — Supplementary Information [file 42003_2025_8384_MOESM2_ESM.pdf]

Supplementary Figure 1. Isolation and differentiation of multiple bone marrow cell populations.

A) Representative images of Alizarin Red and Alkaline Phosphate staining for osteoblast differentiation. B) Representative images of DAPI, Phalloidin, and LipidSpot staining of adipocyte differentiation. Scale bars: 200 μm C) Representative images of DAPI, Phalloidin, and FAP staining during fibroblast differentiation. Scale bars: 100 μm D) Representative images of DAPI, CD163, and TRAP during osteoclast differentiation. Scale bars: 100 μm

Supplementary Figure 2. Stromal cells retain their function and phenotype in longitudinal culture.

A) Quantification of genes specific to osteoblasts, fibroblasts, adipocytes, BMMSCs, and osteoclasts at day 0 and 7 shows either maintenance or increase of gene makers for each cell type. n = 3 experimental replicates B) Representative images and quantification of collagen fiber remodeling (fiber length, fiber width, fiber alignment, and gap area) in prostate cancer seeded devices relative to the control n = 3 experimental replicates. Scale bars: 100 μm Each point indicates a technical replicate. * $p \leq 0.05$, ** $p \leq 0.01$, *** $p \leq 0.005$ (T-Test).

Supplementary Figure 3. Identification of different cell population within the mixed population cluster

A) UMAP plots showing Harmony integration of single-cell RNA sequencing for cluster mixed population at days 4 and 7. B) Feature plots at day 4 and 7 for different marker genes for several cell types: *VWF* for endothelial cells*, ADIPOQ* for adipocytes, *COL1A1* and *BGN* for osteoblast and *SRGN* for immune cells

Supplementary Figure 4. Gating strategy for Prostate Cancer Cell characterization.

Supplementary Figure 5 Clustergram of qPCR results.

Supplementary Figure 6. Representative images from Darolutamide and Docetaxel killing experiments. Representative images of Hoechst and Propidium Iodide (dead cells) staining in DU-145, LAPC4 and LNCaP spheroids after treatment with control (DMSO), 10μM of darolutamide and 40nM of docetaxel in the absence and presence (BoneChip) of the bone microenvironment. Scale bars: 200 μm

Supplementary Figure 7. Fluorescently conjugated ADC diffuses across the MPS within one hour.

A) Representative images of a diffusion assay out of an iPSC EC microvessel (dashed white outline), after SG was conjugated with a fluorophore, at 0 minutes and 60 minutes. B) Quantification of SG diffusion out of the microvessel shows on average 64% of the ADC diffuses out of the vessel within one hour. Scale bars: 400 μm

Supplementary Figure 8. Representative images from SG killing experiments.

A) Representative images of Hoechst and Propidium Iodide (dead cells) staining in primary prostate cancer spheroids from all three donors and at both treatment concentrations shows that killing was evident at 5 and 10 μg/mL in Donor 3 spheroids but only at 10 μg/mL in Donors 1 and 2. B) Representative images of Hoechst and Propidium Iodide staining of the stromal cells (Donor 3) after SG and Isotype ADC treatment at 5 μg/mL shows minimal stromal cell death in all treatment conditions**.** Scale bars: 100 μm

Supplementary Table 1. Composition of the 9 different media formulations screened for MPS co-culture.

Supplementary Figure 1.

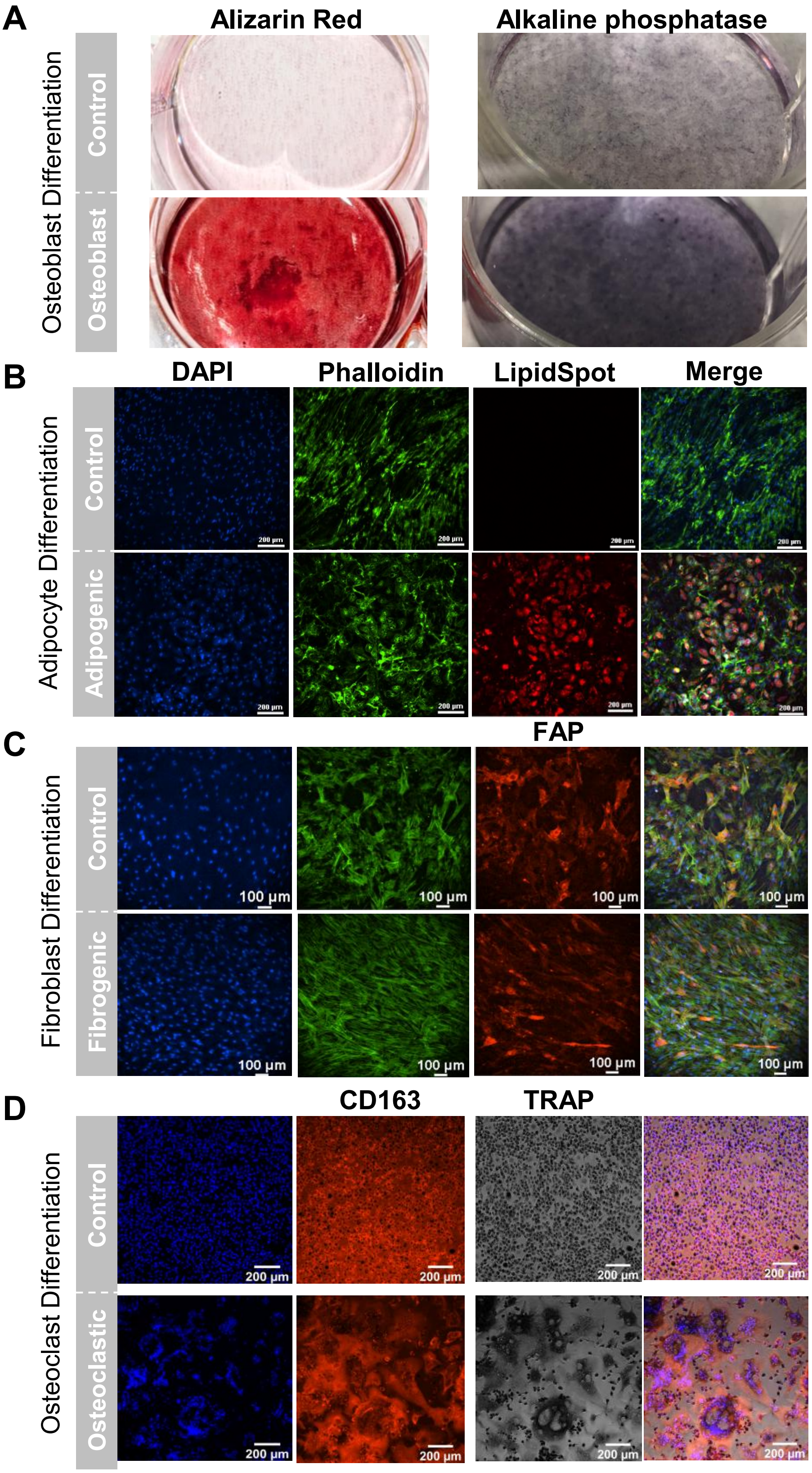

Supplementary Figure 2.

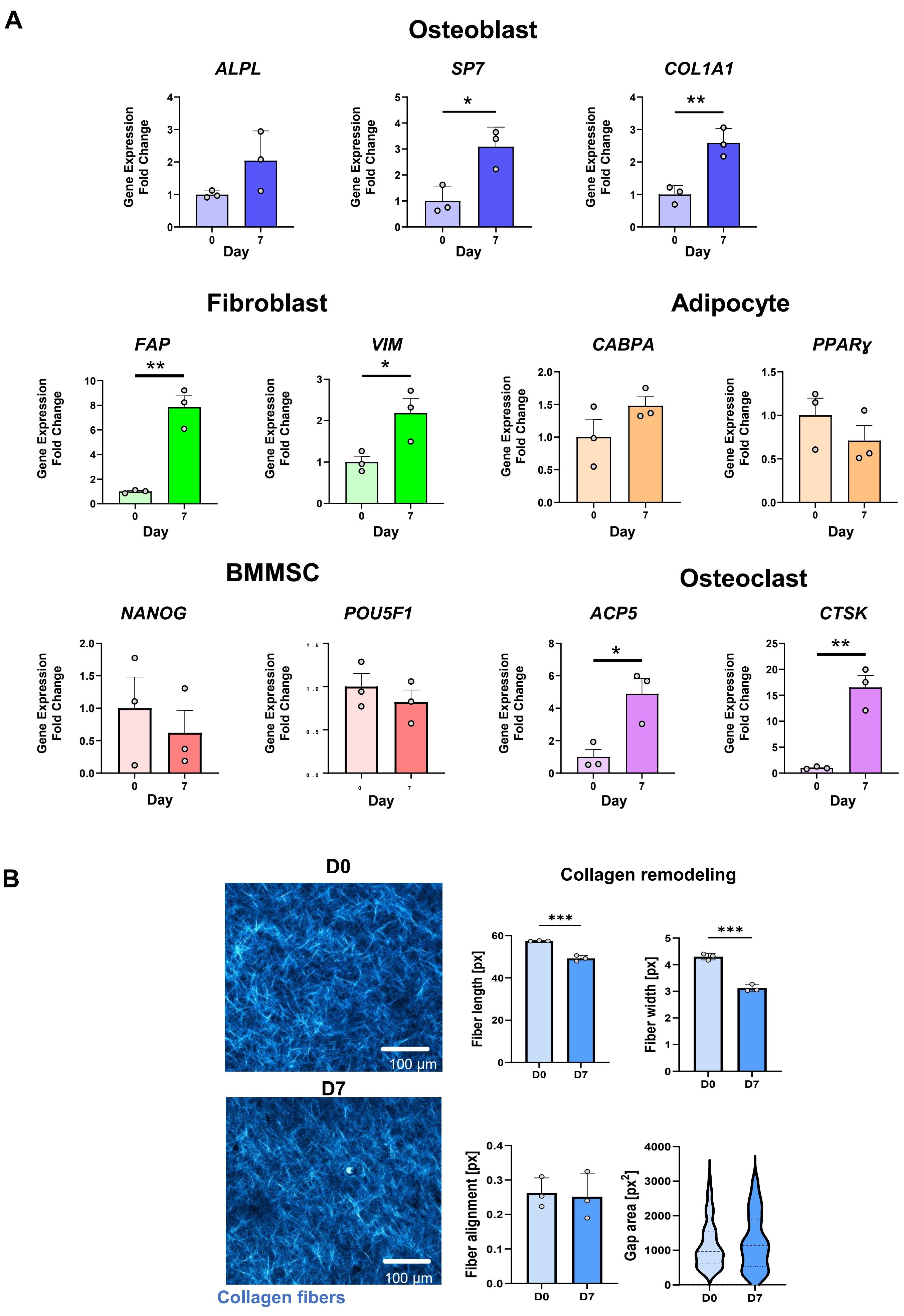

Supplementary Figure 3.

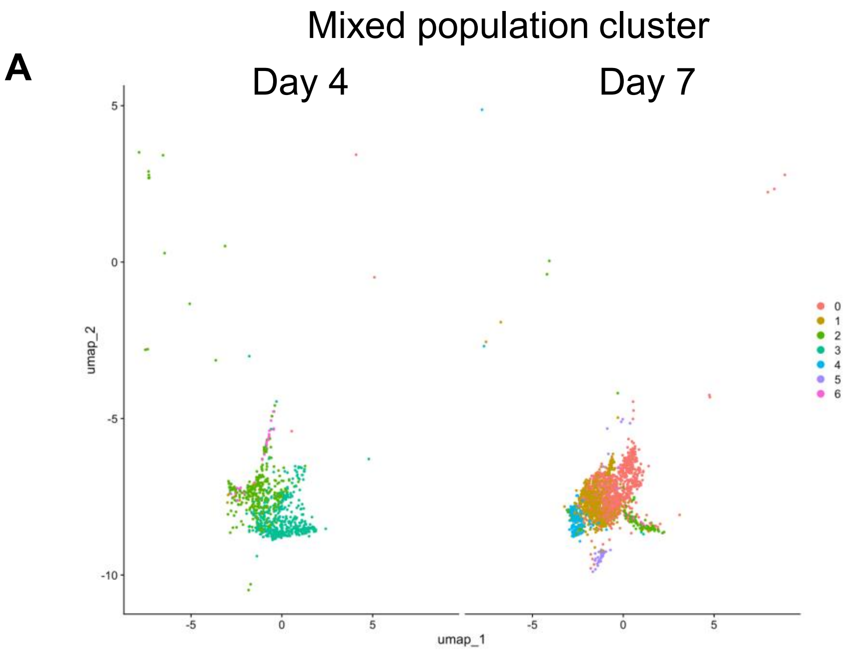

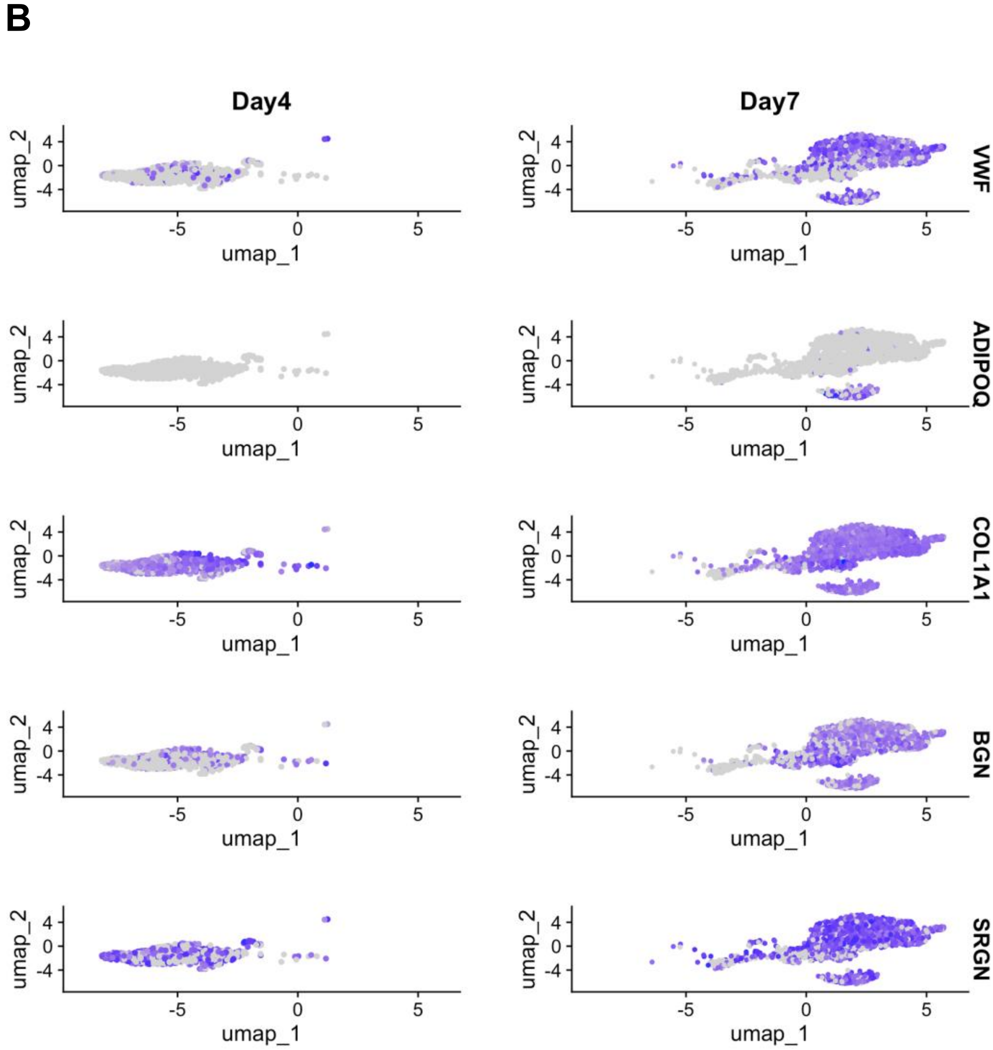

Supplementary Figure 4.

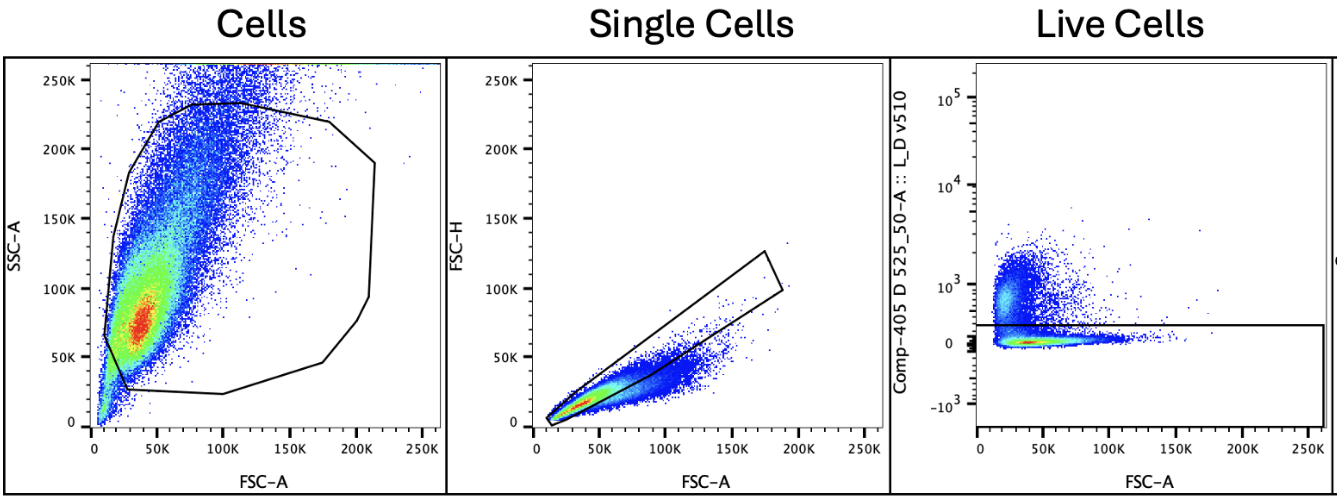

Supplementary Figure 5.

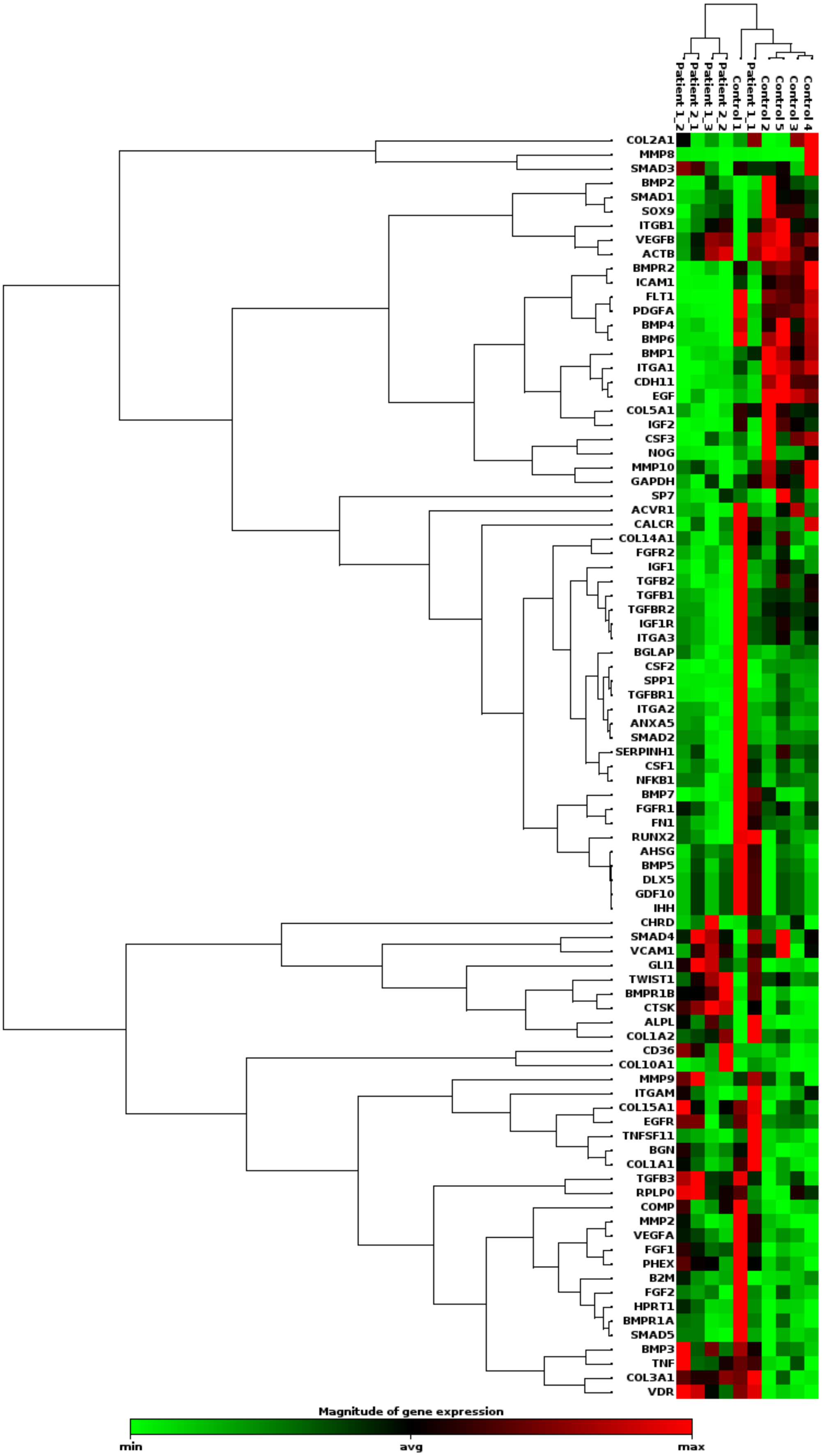

Supplementary Figure 6.

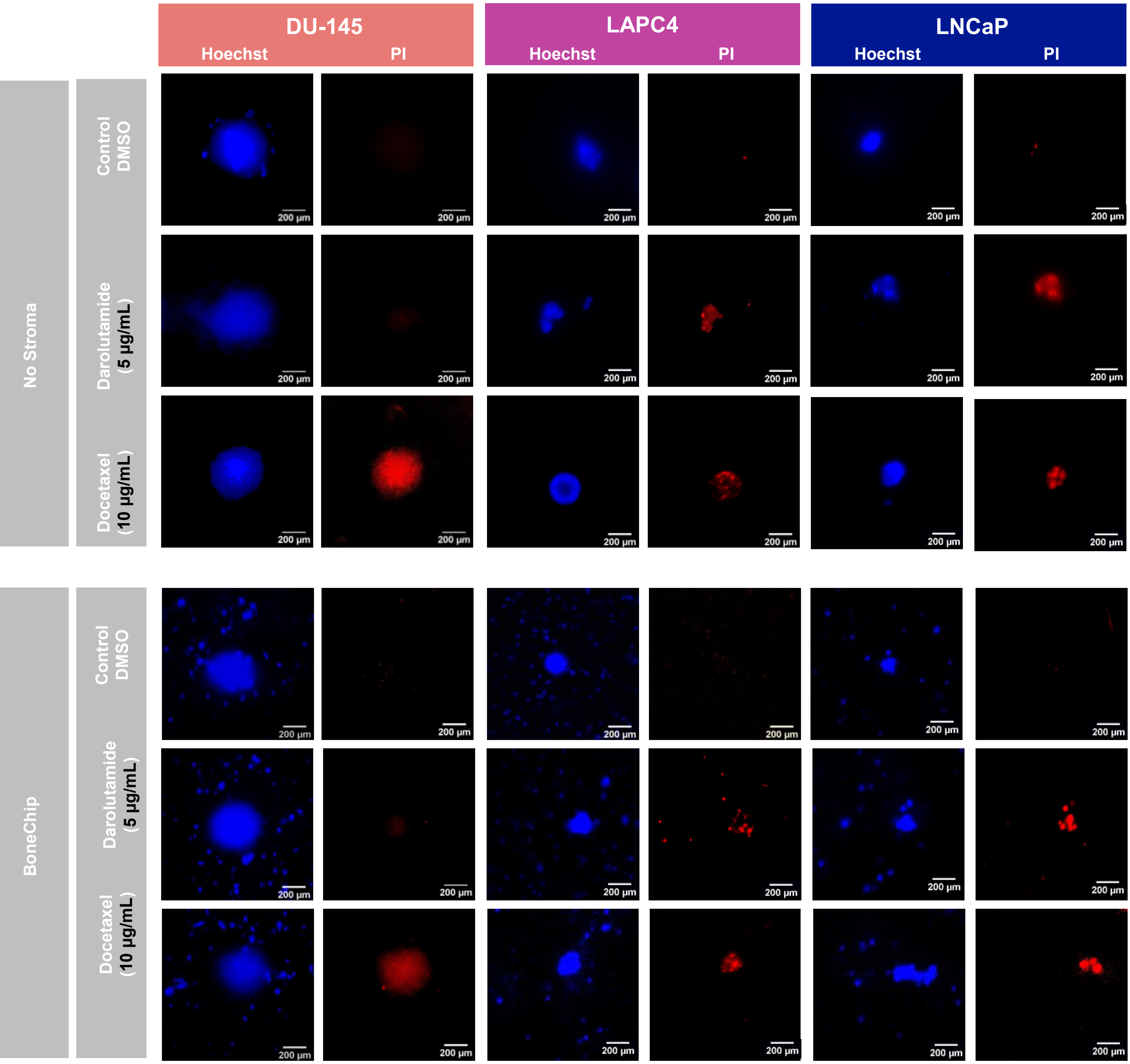

Supplementary Figure 7.

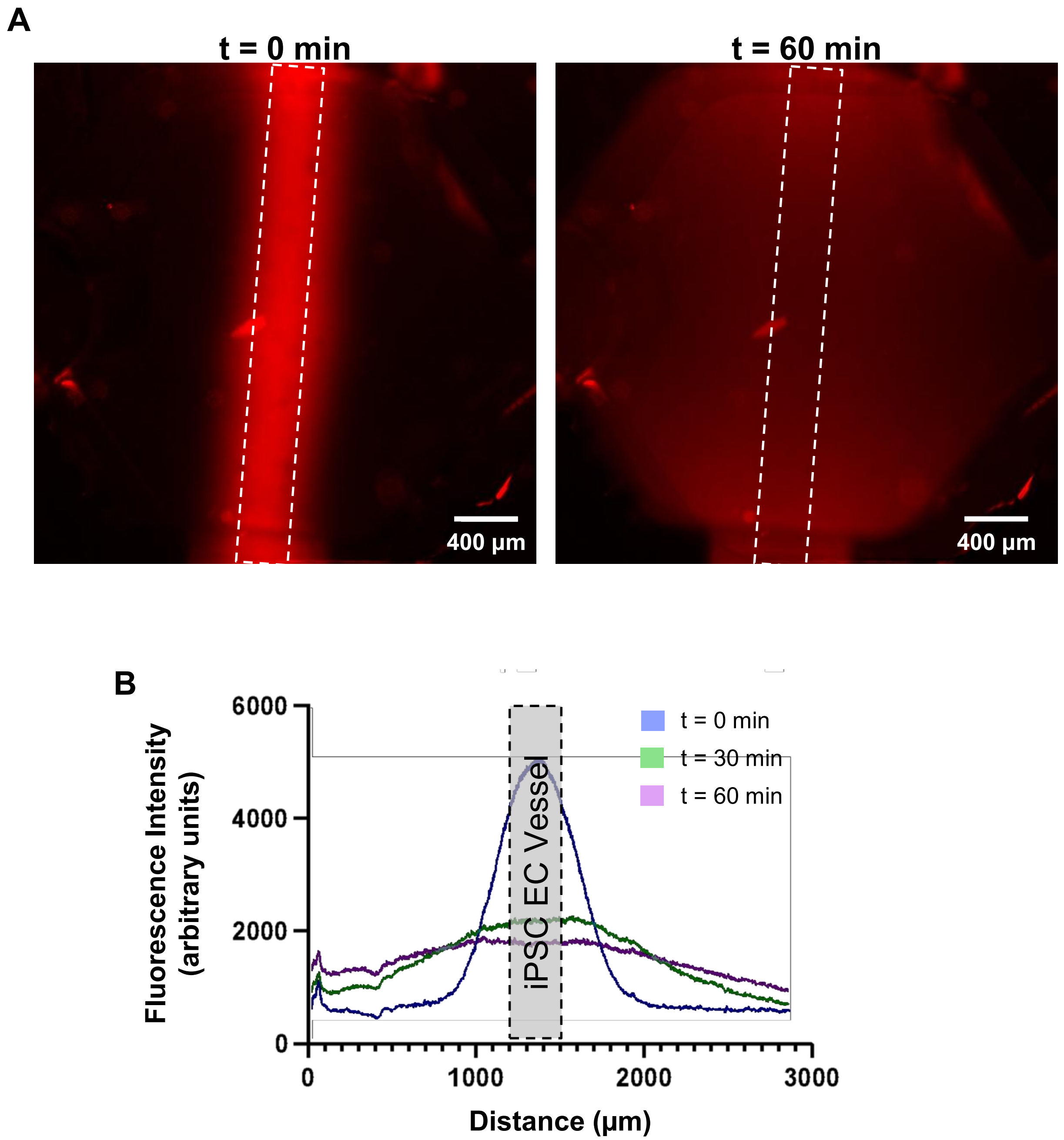

Supplementary Figure 8.

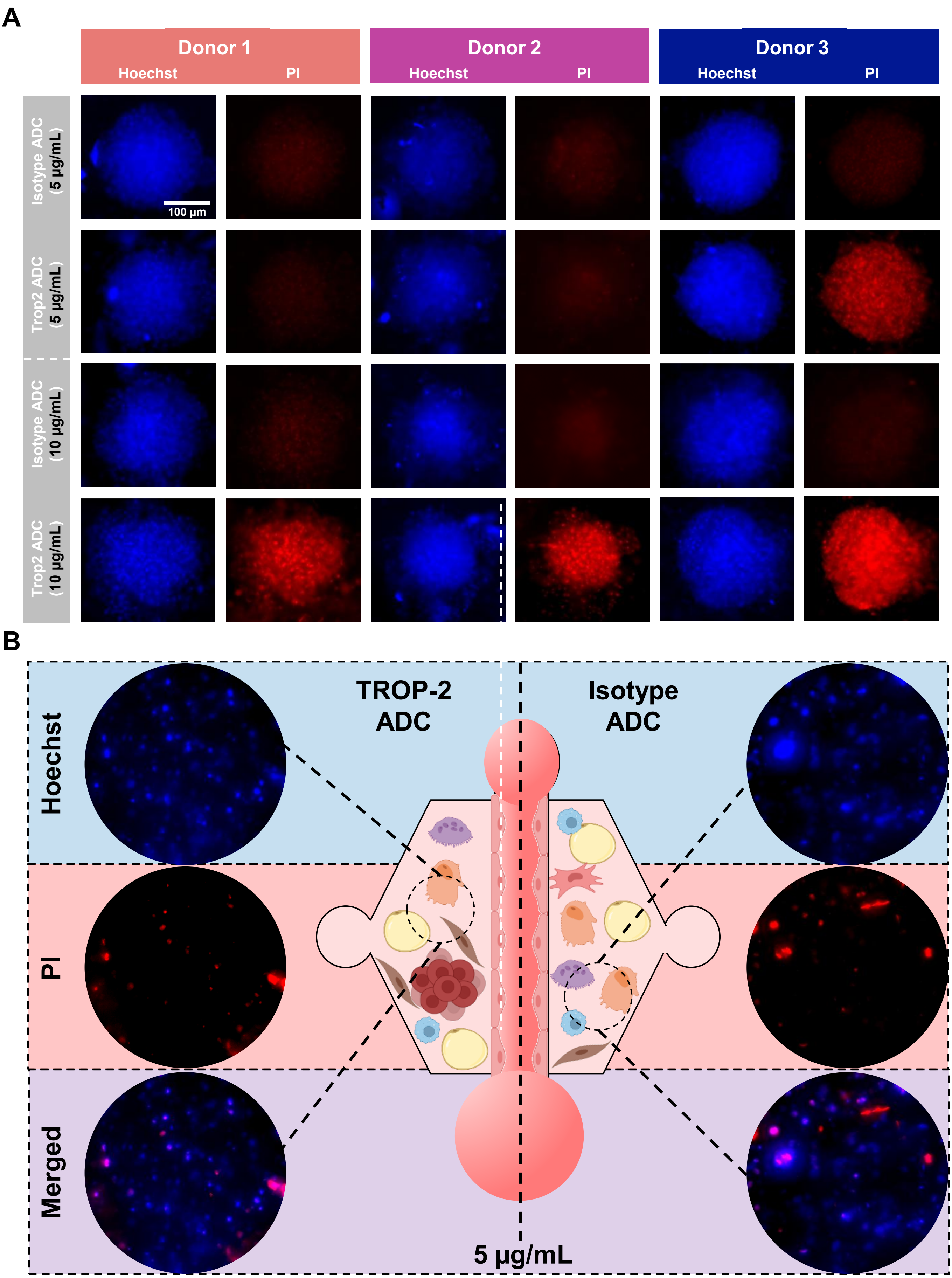

Supplementary Table 1.

**Media 1**	PEpiCM media<br>1% PEpiCGS supplement
**Media 2**	iPSC media<br>10% iCell endothelial cells Medium Supplement<br>5ng/ml rh FGF basic factor<br>50 µg/ml Ascorbic Acid<br>1 µg/ml Hydrocortisone Hemisuccinate<br>15 ng/ml rh IGF-1 factor<br>5 ng/ml rh EGF factor<br>5 ng/ml rh VEGF factor<br>0.75U/mL Heparin Sulfate<br>30mg/ml gentamicin<br>15 µg/ml Amphotericin B.
**Media 3**	PEpiCM media<br>1% PEpiCGS supplement<br>10% iCell endothelial cells Medium Supplement<br>5ng/ml rh FGF basic factor<br>50 µg/ml Ascorbic Acid<br>1 µg/ml Hydrocortisone Hemisuccinate<br>15 ng/ml rh IGF-1 factor<br>5 ng/ml rh EGF factor<br>5 ng/ml rh VEGF factor<br>0.75U/mL Heparin Sulfate<br>30mg/ml gentamicin<br>15 µg/ml Amphotericin B.
**Media 4**	iPSC basal media<br>1% PEpiCGS supplement<br>10% iCell endothelial cells Medium Supplement<br>5ng/ml rh FGF basic factor<br>50 µg/ml Ascorbic Acid<br>1 µg/ml Hydrocortisone Hemisuccinate<br>15 ng/ml rh IGF-1 factor<br>5 ng/ml rh EGF factor<br>5 ng/ml rh VEGF factor<br>0.75U/mL Heparin Sulfate<br>30mg/ml gentamicin<br>15 µg/ml Amphotericin B.
**Media 5**	47% PEpiCM medium<br>47% VascuLife medium<br>0.5% PEpiCGS supplement<br>5% iCell endothelial cells Medium Supplement<br>2.5 ng/ml rh FGF basic factor<br>25 µg/ml Ascorbic Acid<br>0.5 µg/ml Hydrocortisone Hemisuccinate<br>7.5 ng/ml rh IGF-1 factor<br>2.5 ng/ml rh EGF factor<br>2.5 ng/ml rh VEGF factor<br>0.38 U/mL Heparin Sulfate<br>15 mg/ml gentamicin<br>7.5 µg/ml Amphotericin B.
**Media 6**	40% PEpiCM complete<br>40% VascuLife complete<br>1% PEpiCGS supplement<br>10% iCell endothelial cells Medium Supplement<br>5ng/ml rh FGF basic factor<br>50 µg/ml Ascorbic Acid<br>1 µg/ml Hydrocortisone Hemisuccinate<br>15 ng/ml rh IGF-1 factor<br>5 ng/ml rh EGF factor<br>5 ng/ml rh VEGF factor<br>0.75U/mL Heparin Sulfate<br>30mg/ml gentamicin<br>15 µg/ml Amphotericin B.

**Media 7**	Macrophage DXF media<br>50ng/ml M-CSF
**Media 8**	23% PEpiCM medium<br>23% VascuLife medium<br>48% Macrophage DXF media<br>0.5% PEpiCGS supplement<br>5% iCell endothelial cells Medium Supplement<br>2.5 ng/ml rh FGF basic factor<br>25 µg/ml Ascorbic Acid<br>0.5 µg/ml Hydrocortisone Hemisuccinate<br>7.5 ng/ml rh IGF-1 factor<br>2.5 ng/ml rh EGF factor<br>2.5 ng/ml rh VEGF factor<br>0.38 U/mL Heparin Sulfate<br>15 mg/ml gentamicin<br>7.5 µg/ml Amphotericin B.<br>2.5% Promocell DXF supplement
**Media 9**	23% PEpiCM medium<br>23% VascuLife medium<br>48% Macrophage DXF media<br>0.5% PEpiCGS supplement<br>5% iCell endothelial cells Medium Supplement<br>2.5 ng/ml rh FGF basic factor<br>25 µg/ml Ascorbic Acid<br>0.5 µg/ml Hydrocortisone Hemisuccinate<br>7.5 ng/ml rh IGF-1 factor<br>2.5 ng/ml rh EGF factor<br>2.5 ng/ml rh VEGF factor<br>0.38 U/mL Heparin Sulfate<br>15 mg/ml gentamicin<br>7.5 µg/ml Amphotericin B.<br>5% Promocell DXF supplement
