# Supplementary material for: Engineering the bone metastatic prostate cancer niche through a microphysiological system to report patient-specific treatment response
Source: Commun Biol. 2025 Jul 1;8:961. doi: 10.1038/s42003-025-08384-2 (PMC12218145; doi:10.1038/s42003-025-08384-2)
Supplement: Supplementary file 3 — Reporting summary [file 42003_2025_8384_MOESM3_ESM.pdf]

nature portfolio

Corresponding author(s): Sheena C. Kerr, David J Beebe, Joshua Lang

Last updated by author(s): 27/5/25

Reporting Summary

Nature Portfolio wishes to improve the reproducibility of the work that we publish. This form provides structure for consistency and transparency in reporting. For further information on Nature Portfolio policies, see our Editorial Policies and the Editorial Policy Checklist.

Statistics

For all statistical analyses, confirm that the following items are present in the figure legend, table legend, main text, or Methods section.

n/a	Confirmed	
☐	☒	The exact sample size (*n*) for each experimental group/condition, given as a discrete number and unit of measurement
☐	☒	A statement on whether measurements were taken from distinct samples or whether the same sample was measured repeatedly
☐	☒	The statistical test(s) used AND whether they are one- or two-sided<br>*Only common tests should be described solely by name; describe more complex techniques in the Methods section.*
☐	☒	A description of all covariates tested
☐	☒	A description of any assumptions or corrections, such as tests of normality and adjustment for multiple comparisons
☐	☒	A full description of the statistical parameters including central tendency (e.g. means) or other basic estimates (e.g. regression coefficient) AND variation (e.g. standard deviation) or associated estimates of uncertainty (e.g. confidence intervals)
☐	☒	For null hypothesis testing, the test statistic (e.g. *F*, *t*, *r*) with confidence intervals, effect sizes, degrees of freedom and *P* value noted<br>*Give P values as exact values whenever suitable.*
☒	☐	For Bayesian analysis, information on the choice of priors and Markov chain Monte Carlo settings
☒	☐	For hierarchical and complex designs, identification of the appropriate level for tests and full reporting of outcomes
☒	☐	Estimates of effect sizes (e.g. Cohen's *d*, Pearson's *r*), indicating how they were calculated

Our web collection on statistics for biologists contains articles on many of the points above.

Software and code

Policy information about availability of computer code

Data collection	N/A
Data analysis	The code used for single-cell RNA analysis is available on GibHub at the link https://github.com/fauzan-ahmed/CToC.git

For manuscripts utilizing custom algorithms or software that are central to the research but not yet described in published literature, software must be made available to editors and reviewers. We strongly encourage code deposition in a community repository (e.g. GitHub). See the Nature Portfolio guidelines for submitting code & software for further information.

Data

Policy information about availability of data

All manuscripts must include a data availability statement. This statement should provide the following information, where applicable:

- Accession codes, unique identifiers, or web links for publicly available datasets
- A description of any restrictions on data availability
- For clinical datasets or third party data, please ensure that the statement adheres to our policy

The experimental data results that support the findings of this study are available in EveAnalytics database under the study names “Sacituzumab-Govitecan killing in the bone metastasis chip with patient PCa” (https://eve.eveanalytics.com/assays/assaystudy/1464/), “Effect of the stroma on Trop-2 ADC in primary samples” (https://eve.eveanalytics.com/assays/assaystudy/1466/), “Effect of Tro2-ADC on the bone marrow stroma” (https://eve.eveanalytics.com/assays/assaystudy/1467/), “Trop2 ADC PCa Cell lines in Bone Marrow MPS” (https://eve.eveanalytics.com/assays/assaystudy/1463/), “Cell phenotype in multiphenotype

media" (https://eve.eveanalytics.com/assays/assaystudy/1458/), "Phenotype characterization of stroma cells in the bone MPS" (https://eve.eveanalytics.com/assays/assaystudy/1459/), "Protein analysis of bone marrow MPS in presence and absence of Prostate cancer epithelial cells" (https://eve.eveanalytics.com/assays/assaystudy/1460/), "Conventional treatments for prostate cancer on the Bone Marrow MPS" (https://eve.eveanalytics.com/assays/assaystudy/1461/), "The effect of the stroma in effectivity of conventional treatments against prostate cancer" (https://eve.eveanalytics.com/assays/assaystudy/1462/), "Mineralization over time" (https://eve.eveanalytics.com/assays/assaystudy/1448/), "qPCR bone Chips controls vs tumor" (https://eve.eveanalytics.com/assays/assaystudy/1418/) "Collagen remodelling" (https://eve.eveanalytics.com/assays/assaystudy/1457/), "ADC diffusion assay" (https://eve.eveanalytics.com/assays/assaystudy/878/), "Bone marrow microenvironment viability patient 18" (https://eve.eveanalytics.com/assays/assaystudy/675/), "Bone marrow microenvironment viability patient 22" (https://eve.eveanalytics.com/assays/assaystudy/748/), "Media optimization for Prostate Cancer device" (https://eve.eveanalytics.com/assays/assaystudy/643/).
The code used for single-cell RNA analysis is available on GibHub at the link https://github.com/fauzan-ahmed/CToC.git

Single cells data are not publicly available to maintain the protection of patient privacy. Data sharing requests must be submitted to the University of Wisconsin-Madison for review and approval

Research involving human participants, their data, or biological material

Policy information about studies with human participants or human data. See also policy information about sex, gender (identity/presentation), and sexual orientation and race, ethnicity and racism.

Reporting on sex and gender	All human samples were de-identified
Reporting on race, ethnicity, or other socially relevant groupings	All human samples were de-identified
Population characteristics	All human samples were de-identified
Recruitment	Patients with diagnosed Prostate Cancer were recruited.
Ethics oversight	All patients provided written, informed consent under an Institutional Review Board (IRB) approved protocol at the University of Wisconsin-Madison. All ethical regulations relevant to human research participants were followed

Note that full information on the approval of the study protocol must also be provided in the manuscript.

Field-specific reporting

Please select the one below that is the best fit for your research. If you are not sure, read the appropriate sections before making your selection.

☒ Life sciences ☐ Behavioural & social sciences ☐ Ecological, evolutionary & environmental sciences

For a reference copy of the document with all sections, see nature.com/documents/nr-reporting-summary-flat.pdf

Life sciences study design

All studies must disclose on these points even when the disclosure is negative.

Sample size	At least a sample size of three independent experiments was used to ensure the reproducibility of the data
Data exclusions	We did not exclude any data of the analysis.
Replication	Every experiment was conducted three times independently to ensure reproducibility. In the case of gene expression, several genes from the same of similar pathways were analyzed.
Randomization	To ensure reproducibility and reduce bias, cell populations and treatments were randomly distributed across MPS devices
Blinding	Conditions were named randomly with letters. data was analyzed and them matched with its condition to ensure blindness.

Reporting for specific materials, systems and methods

We require information from authors about some types of materials, experimental systems and methods used in many studies. Here, indicate whether each material, system or method listed is relevant to your study. If you are not sure if a list item applies to your research, read the appropriate section before selecting a response.

Materials & experimental systems

n/a	Involved in the study
☐	☒ Antibodies
☐	☒ Eukaryotic cell lines
☒	☐ Palaeontology and archaeology
☒	☐ Animals and other organisms
☒	☐ Clinical data
☒	☐ Dual use research of concern
☒	☐ Plants

Methods

n/a	Involved in the study
☒	☐ ChIP-seq
☐	☒ Flow cytometry
☒	☐ MRI-based neuroimaging

Antibodies

Antibodies used	Sacituzumab govitecan (SG) and isotype ADC were provided by Gilead Sciences.Ghost Dye™ Violet 510 fixable live/dead stain (Tonbo Biosciences, Sand Diego, CA), Fc blocker (Fc Block, BD Biosciences), and fluorescently labeled antibodies including EpCAM Brilliant Violet 650, TROP2 PE, CD49f PerCP Cy5.5, PSMA PE-Cy7 (Biolegend), PSA-Cy5 (Bioss), AR (Cell Signaling) and Donkey Anti-Rabbit Alexa Fluor488 (Biolegend). 5 µg/ml anti-human CD31-CoraLite 594 (Proteintech, L594-11265), 5mg/ml anti-TROP2-488 (R&D, AF650, goat), 5mg/ml Anti-CD163-594 (Novus, BM4041AF594), 5mg/ml anti-RANK-488 (Novus, 64C1385),
Validation	For flow cytometry experiments, antibody validation was performed using fluorescence minus one (FMO) controls, while for microscopy imaging, validation was conducted using secondary antibodies alone, without primary antibodies. All the antibodies has been tested before for flow cytometry or fluorescence microscopy by their manufacturer.

Eukaryotic cell lines

Policy information about cell lines and Sex and Gender in Research

Cell line source(s)	The cell lines DU-145, LNCAP and LAPC4 were directly obtained from ATCC. Monocytes were isolated from peripheral blood draws under approved Institutional Review Board (IRB) protocol. Bone marrow-derived mesenchymal stem cells were isolated from leftover bone marrow filters collected from normal healthy bone marrow donors, based on an IRB-exempt protocol of the University of Wisconsin Bone Marrow Transplant Program. Human prostate tissues were obtained at the University of Wisconsin-Madison from patients with prostate cancer undergoing radical prostatectomy who had received no prior treatments. The University of Wisconsin IRB approved utilization of all the tissue samples in this study and written and informed consents were obtained from all patients.
Authentication	All cell lines were obtained directly from ATCC. MSC markers were evaluated by flow cytometry using CD105, CD73, and CD90, after gating on live, single, cells negative for immune and endothelial markers. MSCs were also evaluated for the ability to differentiate into osteoblasts and adipocytes. The above MSC identification criteria was performed according to guidelines from the MSC committee of the International Stem Cell Society (Dominici, et al). For the primary patient-derived prostate tissue, the prostate cancer diagnosis was confirmed by a pathologist for all samples. Prostate markers were evaluated by flow cytometry using EPCAM, TROP2, CD49f , PSMA and PSA. Macrophages were confirmed by immunofluorescence. The markers used were CD68, CD 163 ad CD206
Mycoplasma contamination	Mycoplasma is tested regularly.
Commonly misidentified lines (See ICLAC register)	This study does not use any commonly misidentified cell lines.

Flow Cytometry

Plots

Confirm that:

☒ The axis labels state the marker and fluorochrome used (e.g. CD4-FITC).

☒ The axis scales are clearly visible. Include numbers along axes only for bottom left plot of group (a 'group' is an analysis of identical markers).

☒ All plots are contour plots with outliers or pseudocolor plots.

☒ A numerical value for number of cells or percentage (with statistics) is provided.

Methodology

Sample preparation	Cells were stained with Ghost Dye™ Violet 510 fixable live/dead stain (Tonbo Biosciences, Sand Diego, CA), Fc blocker (Fc Block, BD Biosciences), and fluorescently labeled antibodies including EpCAM Brilliant Violet 650, TROP2 PE, CD49f PerCP Cy5.5, PSMA PE-Cy7 (Biolegend). For intracellular staining, fixation and permeabilization was performed following the manufacturer's protocol (eBioscience, Thermo Fisher Scientific, MA) followed by staining with PSA-Cy5 (Bioss), AR (Cell Signaling) and Donkey Anti-Rabbit Alexa Fluor488 (Biolegend). Cells were acquired on a BD LSRII instrument (BD Biosciences, Franklin Lake, NJ, USA). Data was analyzed with FlowJo v10.7.1 (FlowJo LLC, by BD Biosciences, Ashland, OR, USA). Gating

controls included Internal Negative Controls (INC) and Fluorescent Minus One (FMO) controls.

Instrument	LSR & Fortessa
Software	FowJo
Cell population abundance	At least 10,000 live cells were used for flow cytometry studies
Gating strategy	Standard FMO were used to establish positive and negative gates

☒ Tick this box to confirm that a figure exemplifying the gating strategy is provided in the Supplementary Information.
